# Associations between cognition, anxiety, depression, and residual dizziness in elderly people with BPPV

**DOI:** 10.3389/fnagi.2023.1208661

**Published:** 2023-08-31

**Authors:** Jin Sun, Xiaobao Ma, Ying Yang, Kuan He, Wei Wang, Jiali Shen, Lu Wang, Xiangping Chen, Yulian Jin, Jun Yang, Jianyong Chen

**Affiliations:** ^1^Department of Otorhinolaryngology-Head and Neck Surgery, Xinhua Hospital Shanghai Jiao Tong University School of Medicine, Shanghai, China; ^2^Shanghai Jiao Tong University School of Medicine Ear Institute, Shanghai, China; ^3^Shanghai Key Laboratory of Translational Medicine on Ear and Nose Diseases, Shanghai, China

**Keywords:** elderly, BPPV, residual dizziness, anxiety, depression, cognition, vertigo

## Abstract

**Objective:**

To investigate the associations between cognition, anxiety, depression, and residual dizziness after successful repositioning maneuvers in the elderly with benign paroxysmal positional vertigo (BPPV).

**Methods:**

We enrolled 40 elderly patients with BPPV in our outpatient department. We used the Dizziness Handicap Inventory (DHI), Visual Analog Scale (VAS), Patient Health Questionnaire-9 (PHQ-9), and Generalized Anxiety Disorder Questionnaire-7 (GAD-7) to assess the degree of dizziness, anxiety, and depression of participants before repositioning therapy, respectively. At the 1-week follow-up after BPPV treatment, each participant will be reassessed and divided into a group with residual dizziness (RD) and a group without residual dizziness (NRD) based on the follow-up DHI score. The Mini-Mental State Examination (MMSE) evaluated the cognitive function of the participants.

**Results:**

The age, gender, duration of BPPV, and involved semicircular canals in the two groups did not show a significant difference. The RD group scored significantly higher on the DHI (*p* = 0.006), GAD-7 (*p* < 0.001), and PHQ-9 (*p* = 0.002) before the repositioning treatment than the NRD group. The two groups had no significant difference in MMSE score (*p* = 0.381). Anxiety and depression scores before repositioning treatment significantly and positively correlated with follow-up DHI scores (*r* = 0.678 and 0.522, respectively), but the MMSE score did not significantly relate to it. The univariate linear regression showed that the DHI (*p* < 0.001), GAD-7 (*p* < 0.001), and PHQ-9 (*p* = 0.002) scores before treatment could predict residual dizziness. The multivariate linear regression showed that GAD-7 before treatment was the only significant predictor of residual dizziness (*p* < 0.001).

**Conclusion:**

The level of dizziness, anxiety, and depression before treatment can predict residual dizziness after successful repositioning maneuvers in the elderly with BPPV. Anxiety may be the strongest predictor of residual dizziness after successful repositioning treatment in elderly BPPV patients.

## 1. Introduction

Benign paroxysmal positional vertigo (BPPV) is the most common cause of recurrent vertigo in the elderly. Nearly 40% of elderly patients seen for dizziness reported having BPPV (Ekvall Hansson et al., 2005). The 1-year prevalence of BPPV increases steeply with age, from 0.5 percent in those under 40 years old to 3.4 percent in those above 60 years old (von Brevern et al., 2007). The cumulative incidence of BPPV reaches almost 10% by age 80 (von Brevern et al., 2007). The typical clinical manifestations of BPPV are recurrent short-duration vertigo and positional nystagmus provoked by changes in the head position relative to gravity (Bhattacharyya et al., 2017). Treatment of geriatric BPPV patients included canalith repositioning procedures (CRPs), the same as CRPs for younger patients.

Some patients reported residual symptoms, such as lightheadedness, dizziness, unsteadiness, and disequilibrium, called residual dizziness, after successful CRPs. The reported prevalence of residual dizziness varied from 31 to 61% (Seok et al., 2008; Teggi et al., 2011, 2013). Patients older than 65 years reported more commonly experienced residual dizziness after BPPV was resolved, with percentages from 34.8 to 36.6% and a mean duration of 13.4 ± 7.5 days (Teggi et al., 2011; Vaduva et al., 2018). Vertigo and residual dizziness put elderly BPPV patients at higher risk for falls and fractures when compared with their healthy peers (Balatsouras et al., 2018). Disabilities caused by vertigo and residual dizziness, and fears of falls make old patients physically inactive and reduce daily activities. Eventually, the health-related quality of life of elderly BPPV patients is impaired (Lopez-Escamez et al., 2005). The age of BPPV onset, duration of vertigo, and times of CRP performed were related to residual dizziness (Vaduva et al., 2018). However, the underlying mechanism of residual dizziness after the resolution of vertigo has not yet been fully understood.

Increasingly, studies reported the potential links between psychological impairment, vertigo, and residual dizziness in BPPV patients. A study showed that nearly half of BPPV patients with a history of psychiatric disorder described residual dizziness (Vaduva et al., 2018). The risk of residual dizziness symptoms was related to self-perceived anxiety and depression in elderly BPPV patients as well (Oghalai et al., 2000; Teggi et al., 2011). Indeed patients with high anxiety showed more enduring dizziness after vertigo and nystagmus had resolved (Bayat et al., 2020). The coexistence of vestibular symptoms, anxiety, and depression might suggest a possible somatopsychic component to residual dizziness in BPPV patients.

In addition, it has been reported that vestibular disorders are associated with cognitive dysfunction (Rizk et al., 2020). Literature showed that individuals with vestibular vertigo had an eightfold increase in the difficulty of concentration or memory (Bigelow et al., 2016). Individuals with vestibular disorders also had more difficulties participating in cognition-relied daily activities than mobility-relied activities (Harun et al., 2015). However, the studies on cognition impairment in the elderly BPPV population and relations with residual dizziness are limited by far.

The study aimed to investigate the associations between cognition function, psychiatric conditions of anxiety and depression, and residual dizziness after successful CRPs in elderly BPPV patients. We used a set of scales to assess the psychiatric conditions of anxiety and depression, perceived degree of vertigo and dizziness, residual dizziness, and cognition function. We compared these variables between patients who exhibited residual dizziness and patients without residual symptoms. We hypothesized that anxiety and depression were associated with residual dizziness after successful CRPs, which could negatively impact cognitive function among the elderly population diagnosed with BPPV. This research will contribute to understanding residual dizziness’s mechanism after successful CRPs and the potential relationships between vestibular conditions and cognition function among the elderly.

## 2. Materials and methods

### 2.1. Subjects

We recruited 68 elderly subjects (older than 65 years) who presented to our vertigo center between September 2022 and March 2023. We used the diagnostic criteria of clinical practice guideline of BPPV released by the American Academy of Otolaryngology-Head and Neck Surgery (AAO-HNS) in 2017 (Bhattacharyya et al., 2017). In all patients, positional vertigo and typical nystagmus (latency, duration, and direction) were triggered through Dix-Hallpike tests and/or Supine Roll Tests. To exclude the central pathologies that cause positional nystagmus, all patients received neurotologic examinations, including spontaneous and gaze-evoked nystagmus, smooth pursuit and ocular saccades, optokinetic nystagmus, and balance assessment. Patients with pathological signs in these tests underwent MRIs. The exclusion criteria were: (1) patients with central nervous system pathologies that caused positional vertigo and nystagmus; (2) patients with other dizziness conditions that cause secondary BPPV, such as Ménière’s disease (MD), vestibular migraines (VM), or vestibular neuritis (VN); (3) Patients had unresolved vertigo and nystagmus after three times repositioning maneuvers at the first visit and the next day. (4) Illiterate patients who were unable to complete the assessments. (5) patients with a history of psychiatric disorders. Eventually, we enrolled 40 patients for analyses; 28 patients were excluded for the absence of the follow-up (*n* = 18) and for not completing all questionnaires (*n* = 10). The sample size meets the minimum requirements of statistical analyses according to the power calculation.

### 2.2. Questionnaires

This study utilized a set of scales to collect relevant data. The Dizziness Handicap Inventory (DHI) and Visual Analog Scale (VAS) were used to assess the degree of dizziness. The DHI comprises 25 items grouped into three dimensions: physical, functional, and emotional (Jacobson and Newman, 1990). The VAS was designed to reflect the perceived vertigo and dizziness in this study. VAS is on a scale of 0–10, with 0 indicating no vertigo and 10 indicating extreme vertigo.

The 7-item Generalized Anxiety Disorder Scale (GAD-7) evaluated the anxiety state. The total score on the GAD-7 is 21, with higher scores representing higher anxiety levels. Scores of 5, 10, and 15 represented cut-off points for mild, moderate, and severe levels of anxiety, respectively (Spitzer et al., 2006). The 9-item Patient Health Questionnaire (PHQ-9) screened the depressive symptom of patients. PHQ-9 scores range from 0 to 27. For the PHQ-9, 5, 10, 15, and 20 cut-off points were interpreted as mild, moderate, moderately severe, and severe depression symptoms, respectively (Bianchi et al., 2022). In this study, the GAD-7 and PHQ-9 were only used to evaluate the anxiety and depressive symptoms of participants. It’s essential to mention that none of the patients were diagnosed by psychiatrists.

The Chinese version of the Mini-Mental State Examination (MMSE) was employed to assess the cognition function of subjects. The MMSE includes the orientation, memory, recall, calculation, and language subcomponents, scoring 30 (Pinto et al., 2019). The MMSE is widely used in cognition assessment and was proven to be sensitive to detecting mild cognition impairment. The lower the score, the more severe the impairment in cognitive function.

### 2.3. Study design

We performed Dix-Hallpike tests and Supine Roll Tests on all patients to diagnose BPPV and determine which semicircular canal (SCC) was affected. In the Dix-Hallpike maneuvers, positional vertigo and up-beating nystagmus with torsional component illustrated posterior semicircular canal BPPV (PSC-BPPV). In the Supine Roll Tests, the presence of geotropic or ageotropic horizontal nystagmus accompanied by positional vertigo indicated horizontal semicircular canal BPPV (HSC-BPPV). Nystagmus was recorded without visual fixation using a wired infrared video-Frenzel goggle connected to G-force™ otolith diagnosis and CRP therapy instrument. Two experienced otolaryngologists made the diagnosis of BPPV. Once the diagnosis was confirmed, we used a set of scales to assess the patients’ dizziness handicap, anxiety, depression, and cognition function before applying CRPs.

We chose the CRPs based on the involved SCC and strongly recommended in the clinical practice guideline by AAO_HNS in 2017. Patients with PSC-BPPV were treated with the Epley maneuver. The Epley maneuver utilizes gravity to transport the free-floating otoliths in the posterior SCC back to the vestibule (Bhattacharyya et al., 2017). Patients with geotropic or ageotropic type of HSC-BPPV were treated with the Barbecue maneuver or Gufoni maneuver, respectively. The Barbecue maneuver was designed to move free-floating otoliths in horizontal SCC and treat the geotropic type of HSC-BPPV. In contrast, the Gufoni maneuver aims to detach otoliths from the cupula and treat the ageotropic type of HSC-BPPV (Bhattacharyya et al., 2017). Two physiotherapists applied the CRPs. The CRPs were performed up to a maximum of three times in a single session.

**FIGURE 1:**
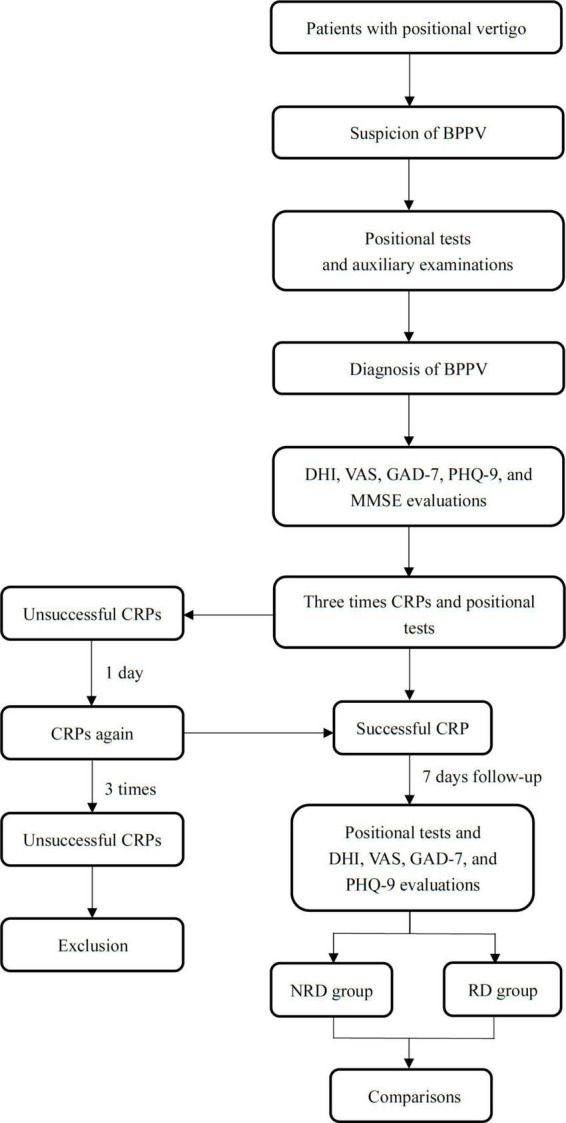
A flow chart describing the research design. BPPV, benign paroxysmal positional vertigo; DHI, dizziness handicap inventory; VAS, visual analog scale; GAD-7 indicates generalized anxiety disorder 7-item scale; PHQ-9, patient health 9-item questionnaire; CRP, canalith repositioning procedure.

Two doctors applied positional tests for patients after the CRPs. The complete remission of subjective vertigo and objective positional nystagmus was the criteria for successful CRPs treatment. Patients attended a 1-week follow-up after the resolution and received evaluations of residual dizziness, anxiety, and depression. The cognition function of patients was only evaluated at the first presentation due to the stability of cognition within 1 week (Fu et al., 2021; Lee et al., 2022). The patients were divided into the no residual dizziness (NRD) group and the residual dizziness (RD) group based on the follow-up DHI scores. A flow chart showing the study design is shown in Figure 1. The studies involving human participants were reviewed and approved by the Ethics Committee of Xinhua Hospital Affiliated with Shanghai Jiao Tong University School of Medicine (Approval No. XHEC-D-2017-046). All patients were informed in detail about the purpose and method of the study, and written informed consent was obtained from all patients.

### 2.4. Statistical analysis

We used a Chi-square test to study the demographics in the two groups of BPPV patients. The normality of DHI, VAS, MMSE, GAD-7, and PHQ-9 scores of the two groups was analyzed by the Shapiro-Wilk test. The study measures were normally distributed except for the GAD-7 and PHQ-9 scores of the NRD group at pre- and post-treatment assessment and VAS ratings of two groups at post-treatment assessment. An unpaired t-test was applied to analyze the differences in cognition function and DHI scores between the NRD and RD groups. All data meet the homogeneity of variance. We performed the non-parametric Mann-Whitney *U*-test to study the differences in anxiety and depression symptoms between the NRD and RD groups. Pearson’s correlation test was applied for the correlation between variables. Significance was determined at *p* < 0.05, and all statistical analyses were performed by SPSS 23 (SPSS Inc, Chicago, IL, USA). The power calculation was conducted by G*Power 3.1 (Heinrich-Heine-Universität Düsseldorf). The power was above 90% in correlation, *t*-tests, and linear regression tests.

## 3. Results

### 3.1. Demographics

Forty patients met all criteria in all recruited subjects. The average age of the cohort was 69.60 ± 4.06 years. Of the 40 patients, 28 were female (the gender ratio was 2.33:1), consistent with the prevalence characteristics of BPPV (Neuhauser and Lempert, 2009). The average duration of symptoms for the entire cohort at the time of presentation was 16.94 days, and the median (P25, P75) was 7.5 (5, 23) days. The average years of education for all included patients was 8.91 ± 1.96 years.

The 40 patients were divided into two groups with 20 cases in each group based on the follow-up DHI scores, namely the NRD group (DHI < 16) and RD group (DHI ≥ 16). Table 1 shows the demographics and results of Chi-square tests in the two groups.

**TABLE 1:** Overall description of the demographics of the two groups.

	NRD	RD	*p*-value
Gender (women: men)	14:6	15:5	0.723
Age, mean (SD)	69.47 (3.62)	69.73 (4.57)	0.860
**Duration of symptoms (days)**			
Mean (SD)	15.81 (21.46)	18.06 (19.76)	0.760
Median	8	7	
Education (years)	9.06 (2.21)	8.75 (1.73)	0.659
**Semicircular canal, *n* (%)**			
Posterior	19 (95%)	17 (85%)	0.292
Horizontal	1 (5%)	3 (15%)	
Right	15 (75%)	11 (55%)	0.185
Left	5 (15%)	9 (45%)	
**Type of BPPV, *n***			
Canalolithiasis	20	18	0.147
Cupulolithiasis	0	2	
Recurrence	2 (10%)	3 (15%)	0.633

### 3.2. Dizziness handicap inventory

In the pre-treatment assessment, the NRD group had a DHI mean total score of 33.38 ± 21.09, which were significantly different from the mean total score in the RD group of 55.5 ± 20.82 (*t* = −2.987, *p* = 0.006, effect size = 1.055). Figure 1 compares three DHI subscales between the groups in two assessments. Broken down by subscale, there were statistically significant differences between NRD and RD groups in DHI-P (*t* = −2.297, *p* = 0.029), DHI-F (*t* = −2.547, *p* = 0.016), and DHI-E (*t* = −2.809, *p* = 0.009) scores. The RD group had a mean DHI-E score of 16.63 ± 9.40, more than twice the DHI-E score of 7.88 ± 8.18 in the NRD group.

**FIGURE 2:**
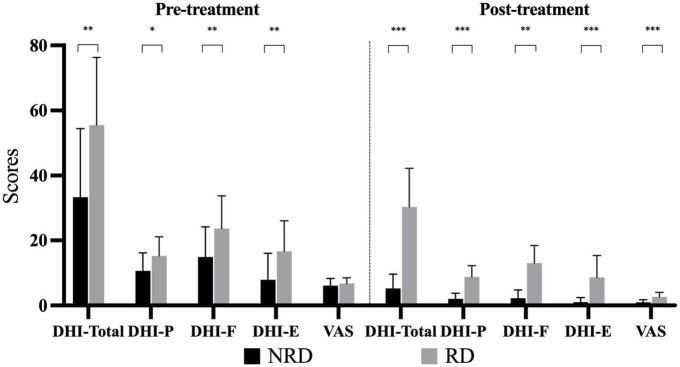
Bar chart depicting the DHI subscales and VAS scores of two groups at pre- and post-treatments. *p-value < 0.05; **p-value < 0.01; ***p-value < 0.001. DHI-P indicates physical subscale; DHI-F, functional subscale; DHI-E, emotional subscale; VAS, visual analog scale; RD, residual dizziness; NRD, no residual dizziness.

At follow-up, the total score of the RD group decreased to 30.38 ± 11.87, which was significantly higher than the total score of the NRD group at 5.25 ± 4.37 (*t* = −7.944, *p* < 0.001, effect size = 2.809). This difference in DHI scores between the two groups was the principle of grouping. As is shown in Figure 2, the RD group had a significantly higher score than the NRD group in DHI-P (*t* = −6.880, *p* < 0.001), DHI-F (*t* = −7.147, *p* < 0.001), and DHI-E (*t* = −4.434, *p* < 0.001).

### 3.3. Visual analog scale

Figure 2 compares the VAS rating of the two groups at the first visit and follow-up. VAS ratings for vertigo of the two groups (NRD 6.13 ± 2.19, RD 6.75 ± 1.77) did not significantly differ (*t* = −0.889, *p* = 0.381) at the first presentation. The VAS ratings of the two groups were non-normally distributed, with the median (P25, P75) of the NRD group being 1 (0, 2) and that of the RD group being 2 (2, 3) at the follow-up. The Mann-Whitney *U*-test showed that the RD group had a significantly higher VAS rating than the NRD group (*Z* = −3.671, *p* < 0.001, effect size = 1.451) at follow-up.

### 3.4. Mini-mental state examination

**FIGURE 3:**
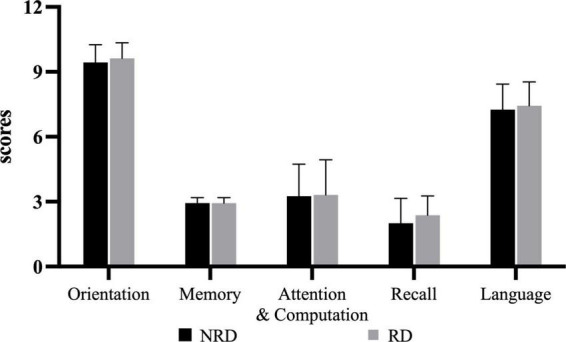
Five categories of MMSE for two groups. RD, residual dizziness; NRD, no residual dizziness.

The mean MMSE total score for the NRD group was 24.88 ± 3.28 and for the RD group was 25.69 ± 2.70 out of a possible 30 points. The unpaired *t*-test showed no significant difference in MMSE total score between the two groups (*t* = −0.764, *p* = 0.451, effect size = 0.270). Figure 3 shows the comparisons in the five subcomponents of the cognition function defined by MMSE. There was also no statistically significant difference between the two groups on each subcomponent.

### 3.5. Generalized anxiety disorder 7-item scale

**FIGURE 4:**
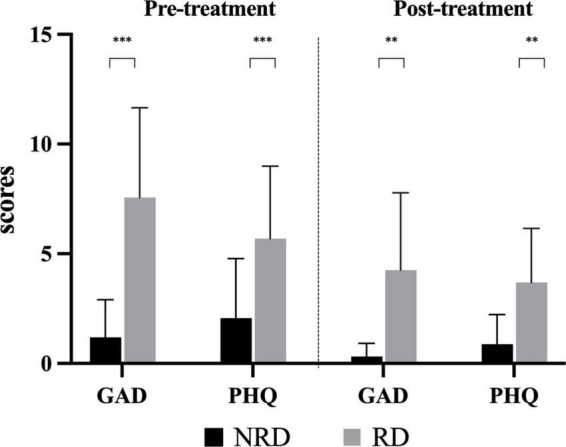
Comparison of the degree of anxiety and depression in two groups at pre- and post-treatment assessments. **p-value < 0.01; ***p-value < 0.001. RD, residual dizziness; NRD, no residual dizziness.

Figure 4 shows the comparisons of GAD-7 and PHQ-9 between groups in two assessments. In the pre-treatment assessment, the median (P25, P75) GAD-7 score for the NRD group was 0 (0, 2) and 8.5 (3.5, 11) for the RD group. The Mann-Whitney *U*-test showed that the GAD-7 score of the NRD group was significantly lower than that of the RD group at (*Z* = −4.229, *p* < 0.001, effect size = 2.027). At follow-up evaluation, The NRD group had a median (P25, P75) GAD-7 score of 0 (0, 0), which was significantly lower than the GAD-7 score of 1 (0, 3) in the RD group as well (*Z* = −3.955, *p* < 0.001, effect size = 1.556).

### 3.6. Patient health 9-item questionnaire

At the first presentation, the NRD group had a median (P25, P75) PHQ-9 score of 1 (0, 4.5) and the RD group had a median (P25, P75) PHQ-9 score of 5 (3.25, 8). The Mann-Whitney *U*-test showed that the PHQ-9 score of the NRD group was significantly lower than that of the RD group (*Z* = −3.101, *p* = 0.002, effect size = 1.200). At the follow-up assessment, PHQ-9 scores significantly differed between the two groups (*Z* = −3.285, *p* = 0.002, effect size = 1.410). The RD group scored 3.5 (1.25, 6), and the NRD group scored 0.5 (0, 1), see Figure 4.

### 3.7. Correlations

We performed Person’s correlation method to analyze the relationship between residual dizziness surveys and cognition, anxiety, and depression scores at the first presentation in all patients. Table 2 shows all correlations. The results showed that the pre-treatment anxiety and depression scores moderately correlated with the follow-up VAS rating (coefficients were 0.381, *p* = 0.032, and 0.497, *p* = 0.004) and highly correlated to the follow-up DHI-P, DHI-F, DHI-E, and total scores. While age, duration of symptom, and MMSE subcomponents did not correlate with the other variables.

**TABLE 2:** Correlations between surveys in all patients.

	VAS- post	DHI-P post	DHI-F post	DHI-E post	DHI total score
Age	0.069	0.235	0.273	0.295	0.305
Duration of symptom	-0.17	0.081	-0.041	-0.098	-0.034
**MMSE**					
Orientation	0.317	-0.033	0.002	-0.343	-0.144
Memory	-0.04	0.022	-0.052	-0.051	-0.037
Attention and computation	0.147	-0.194	-0.125	-0.196	-0.188
Recall	0.051	0.127	-0.017	-0.076	-0.002
Language	0.028	-0.112	0.034	-0.107	-0.059
Total score	0.181	-0.104	-0.061	-0.258	-0.159
GAD-7	0.381*	0.634***	0.587***	0.599***	0.678***
PHQ-9	0.497**	0.596***	0.462**	0.373*	0.522**

*p < 0.05; **p < 0.01; ***p < 0.001.

MMSE, mini-mental state examination; GAD-7, generalized anxiety disorder 7-item scale; PHQ-9, patient health 9-item questionnaire; DHI, dizziness handicap inventory; DHI-P, physical subscale; DHI-F, functional subscale; DHI-E, emotional subscale; VAS-post, visual analog scale at follow-up.

### 3.8. Regression models for predictors of residual dizziness

We applied univariate regression to find predictors of residual dizziness. The follow-up DHI score was used as the dependent variable. The duration of BPPV, the DHI score, the GAD-7 score, and the PHQ-9 score before treatment was chosen as independent variables based on the potential effects on residual dizziness. Table 3 presents the results of the univariate regression. The DHI score, GAD-7 score, and PHQ-9 score before CRPs treatments were significant predictors. We also performed multivariate regression to compare these significant predictors’ effect sizes and relationships. There was no multicollinearity among the independent variables. As presented in Table 3, GAD-7 was the only significant predictor in the model. The *R*^2^ of the model was 0.511, which means that these independent variables account for 51.1% of the variability in DHI score at follow-up.

**TABLE 3:** Linear regression models of DHI score for all patients.

	Unstandardized coefficient	*SE*	*p*	*R* ^2^	Model *p*
Duration	-0.026	0.139	0.852	0.001	0.835
DHI-pre	0.390***	-0.097	<0.001	0.349	<0.001
GAD-7	2.346***	0.465	<0.001	0.459	<0.001
PHQ-9	2.312**	0.690	0.002	0.272	0.002
Multivariate regression				0.511	0.000
DHI-pre	0.186	0.111	0.106		
GAD-7	1.692*	0.709	0.024		
PHQ-9	0.116	0.847	0.892		

*p < 0.05; **p < 0.001; ***p < 0.001.

DHI-pre, dizziness handicap inventory at pre-treatment; GAD-7, generalized anxiety disorder 7-item scale; PHQ-9, patient health 9-item questionnaire.

## 4. Discussion

Our study investigated the potential factors related to residual dizziness after successful CRPs in elderly BPPV patients. Our study found that the age, gender, involved canal, and duration of vertigo did not correlate with the residual dizziness handicaps after successful repositioning maneuvers. These results support previous findings that gender and involved canal were not linked to residual dizziness but disagree with the reported correlation between age and residual dizziness (Vaduva et al., 2018). The variations in population demographics could explain the inconsistency. The prior study included participants of all ages, with half of the participants above 65 years old, and found that patients older than 65 presented a higher percentage of residual dizziness. We only enrolled patients older than 65 years (mean 69.56 ± 3.93 years), and the percentage of residual symptoms in this study was similar to this of participants above 65 years old in the previous study. We thought that the discrepancy in the age span of participants led to different conclusions. The association between the duration of BPPV and residual dizziness was still in dispute. Many researchers had reported that a longer duration of vertigo before diagnosis was linked to the presence of residual dizziness and unsteadiness after the resolution of BPPV (Stambolieva and Angov, 2006; Teggi et al., 2011; Faralli et al., 2016). However, some researchers reported that the duration of BPPV may not be a risk factor for residual symptoms (Kim and Lee, 2014; Giommetti et al., 2017). The age range and sample size of previous studies were different. The different inclusion criteria and relatively small sample size may explain the discrepancy. Our findings corroborate that the duration of BPPV was not related to the presence of residual dizziness in the elderly.

### 4.1. Assessment of dizziness

Our study found that at the time of diagnosis, the DHI subscales scores of the RD group were significantly higher than those of the NRD group, and linear regression analysis demonstrated that the DHI total score before CRPs was a predictor for residual dizziness. Our results accord with previous studies reporting that high DHI subscale scores and total scores at the first presentation are sensitive factors related to the incidence of residual dizziness (Ke et al., 2022; Fu et al., 2023). Our study further confirmed that DHI could be a helpful inventory before treatment to estimate the risk of residual dizziness after successful CRPs and quantify the dizziness handicaps on the quality of life in patients over 65 years old. In this study, we employed VAS to assess the subjective perception of vertigo and dizziness. The VAS rating showed a significant difference among the two groups at follow-up, supporting the opinion that VAS could be a sensitive tool to assess residual dizziness in BPPV patients (Fu et al., 2023). Recently, VAS is getting popular in the realm of vestibular symptoms. Our study shows the advantages of VAS for elderly BPPV patients in terms of being easy to use and overcoming cultural and cognitive barriers.

### 4.2. Cognitive dysfunction and residual dizziness

Previous studies found that patients with bilateral vestibular dysfunction had deficits in visuospatial tasks, navigation, attention, and memory (Brandt et al., 2017), especially in elderly subjects (Caixeta et al., 2012). Even patients with unilateral vestibular impaired performed worse in cognitive domains of visuospatial and navigational tasks (Guidetti et al., 2008; Popp et al., 2017). However, we found that all five categories and the total score of MMSE did not correlate with the follow-up DHI and VAS scores, and the NRD and RD groups presented no differences in the performance of MMSE tasks. Possible reasons for these results are: First, the disease characteristics of BPPV are temporary and self-limited, which may not lead to permanent cognitive impairment. The duration of BPPV of subjects in our study was relatively short. The average duration of all patients was 16.94 ± 20.33 days, with 21 patients (52.5%) diagnosed within 7 days. A study demonstrated that the duration of vestibular symptoms and certain etiologies are more curial in cognitive dysfunction (Rizk et al., 2020). Our results were consistent with previous studies reporting that compared with other chronic vestibular disorders such as MD, VM, and persistent postural-perceptual dizziness (PPPD), BPPV patients did not present vestibular-related cognition disabilities (Liu et al., 2019; Rizk et al., 2020). Second, the MMSE may not thoroughly reflect the cognitive impairment related to vertigo and dizziness in elderly BPPV patients. The cognitive function most closely associated with vestibular disorders is the visuospatial ability, which includes spatial memory, navigation, mental rotation, and mental representation of 3-D space (Bigelow and Agrawal, 2015). In addition, visuospatial functions share the same cortical networks with the vestibular system (Hitier et al., 2014; Previc et al., 2014), and researchers hypothesized that this is one of the underlying mechanisms of vestibular-related cognition dysfunction. However, only one item, namely copying intersecting pentagons, is concerned with visuospatial ability in MMSE, which may not allow for a comprehensive examination of cognition in old patients with BPPV.

### 4.3. Psychiatric disorder and residual dizziness

The most noticeable finding of our study is the relationship between psychiatric symptoms and residual dizziness in old patients with BPPV. We found that the degree of anxiety and depression before treatment was positively correlated with residual dizziness, which accords with previous studies reporting the correlation between the psychological condition and residual dizziness in BPPV patients (Teggi et al., 2011). In addition, the findings that anxiety and depression are significant predictors for residual dizziness and that anxiety is the strongest predictor further demonstrate that anxiety and depression disorder influence the presence of residual dizziness after successful CRPs.

The close association between dizziness or vertigo and psychosomatic disorders such as depression, generalized anxiety disorder, somatization disorders, and panic disorder was well-established (Balaban and Jacob, 2001; Eckhardt-Henn et al., 2008). There is research reported that nearly half of BPPV patients with a psychiatric history presented residual dizziness after repositioning maneuvers (Vaduva et al., 2018), and the incidence of psychiatric disorder raised 5–15 times in patients with vestibular disorders (Pollak et al., 2003). The coexistence of vestibular and psychiatric disorders may imply a potential overlapped neural circuit. This concept was further supported by anatomical and functional links between the vestibular system and structures involved in the pathogenesis of panic disorder or the regulation of fear responses, such as the brainstem blue spot and the nucleus accumbens (Nishiike et al., 2001; Staab et al., 2002; Best et al., 2009). Anxiety and depression have been shown to play an important role in dizziness as a somatic form of the disorder and can be caused by a few stressful events (Wei et al., 2018). Violent episodes of episodic vertigo in patients with BPPV are one such stressful event (Martellucci et al., 2016). When an episode of BPPV is followed by abnormal stimulation of the vestibular system, it can lead to changes in mood, such as tension and anxiety. It has been shown that anxiety-related arousal and hyperventilation increase the effects on various vestibular laboratory parameters, which in turn leads to vestibular dysfunction (Nishiike et al., 2001), causing RD in some patients after CRPs (Ke et al., 2022). This vicious cycle could eventually impair the quality of patients’ life.

We found that the degree of anxiety and depression of the RD group was significantly higher than the NRD group at the first visit. This may be due to acute vertigo episodes triggered or exacerbated the anxiety and depression in elderly BPPV Patients. Kalderon et al. (2022) also proved that the anxiety of patients with BPPV is not a personality trait (trait anxiety) but a temporary state (state anxiety) that is provoked by acute vertigo episodes. Additionally, Jung et al. (2012) reported that a low dose of anxiolytics (etizolam) could alleviate the residual dizziness in BPPV patients, especially for functional and emotional dizziness handicaps. Even if there is no rigorous scientific evidence to generalize this medication in clinical practice, the positive effect may somewhat explain the role of psychiatric disorders in residual dizziness.

In conclusion, anxiety and depression contribute to the residual dizziness of elderly BPPV patients. It is important to incorporate psychological counseling and ongoing monitoring to manage residual dizziness in this patient population.

### 4.4. Limitations and future directions

While our study did yield valuable results, there are still limitations that need to be acknowledged. First, the evaluation tool for cognition dysfunction in elderly BPPV patients we used is not designed to assess the vestibular-related cognition impairment, which could not thoroughly reflect the visuospatial abilities of subjects. According to reports, the Neuropsychological Vertigo Inventory (NVI) can measure cognitive dysfunction in patients experiencing dizziness. Additionally, the objective P300 Event-Related Potential (ERP) can evaluate cognitive impairment that may not be reflected in subjective assessments (Liu et al., 2019; Toyoshima et al., 2020). Therefore, in future studies, we can use the NVI in combination with objective P300 ERP to target vestibular-related cognition impairment in elderly patients with BPPV. Second, the residual dizziness experienced by the elderly participants in this study had a relatively short duration. As a result, it may be unlikely to impact their cognitive function negatively. The future direction is to extend the follow-up period to 3 months and assess the cognition function each month to investigate the effect of residual dizziness on cognition comprehensively. Furthermore, we did not investigate the association between a history of BPPV and residual dizziness due to the relatively small sample size. Future research will expand the sample size and divide the participants according to the history of BPPV to investigate this potential relationship.

## 5. Conclusion

In elderly patients with BPPV, there is a positive correlation between the degree of residual dizziness and the scores of DHI, GAD-7, and PHQ-9 at the first presentation. Vertigo and residual dizziness do not correlate cognition dysfunction in this study. The DHI, GAD-7, and PHQ-9 scores at the first presentation can predict residual dizziness. Among these factors, GAD-7 is the strongest predictor for elderly BPPV patients. The results of this study suggest that it is important to provide psychological support for elderly BPPV patients in managing residual dizziness. Doctors can clarify the mechanisms of RD after successful CRPs to alleviate anxiety and depression among elderly patients. Collaborating with psychiatrists on medication may also contribute to managing residual symptoms after CRPs in elderly BPPV patients.

## Data availability statement

The raw data supporting the conclusions of this article will be made available by the authors, without undue reservation.

## Ethics statement

The studies involving human participants were reviewed and approved by the Ethics Committee of Xinhua Hospital Affiliated with Shanghai Jiao Tong University School of Medicine. The patients/participants provided their written informed consent to participate in this study.

## Author contributions

JS responsible for CRPs application, data collection, and manuscript composition. XM and YY collected the clinical data. KH helped with CRPs practice. WW, JLS, and LW contributed to the data analysis. XC contributed to statistical consultation. JY, YLJ, and JYC responsible for the research design and manuscript revision. All authors contributed to the article and approved the submitted version.
